# Checkpoint blockade inhibitors enhances the effectiveness of a *Listeria monocytogenes*-based melanoma vaccine

**DOI:** 10.18632/oncotarget.27490

**Published:** 2020-02-18

**Authors:** Ryan P. Gilley, Peter H. Dube

**Affiliations:** ^1^ Department of Microbiology, Immunology and Molecular Genetics, The University of Texas Health Science Center at San Antonio, San Antonio, TX 78229, United States

**Keywords:** cancer immunotherapy, cancer vaccination, checkpoint blockade, melanoma, listeria

## Abstract

Melanoma continues to be a significant health concern worldwide despite recent improvements in treatment. Unlike many other prominent cancers, melanoma incidence in both men and women increased over the past decade in the U. S. and much of the developed world. The single greatest risk factor for melanoma is damage from ultraviolet radiation associated with lifestyle. The lifestyle component suggests that although melanoma risk can be minimized with behavioral changes, vaccinating high-risk individuals against melanoma may be the most efficacious preventative method. Accordingly, using a highly attenuated, double-mutant *L. monocytogenes* strain expressing a tumor-associated antigen, we obtained significant protection against melanoma in a mouse model. The *Listeria*-based vaccine induced protection through antigen-specific CD8+ T-cells inducing both a protective primary and a memory T-cell response. Vaccinated animals were significantly protected from melanoma. When used in conjunction with checkpoint blockade treatment, the vaccine substantially reduced tumor size and number relative to animals receiving checkpoint blockade (CPB) alone. This study provides evidence that CPB treatment synergizes with a *L. monocytogenes*-based melanoma vaccine to enhance vaccine-mediated protection.

## INTRODUCTION

The incidence and mortality rates for numerous common human cancers such as breast, cervical, colorectal, lung, ovarian, and prostate have declined over the past fifteen years [1]. Advances in detection, treatment, and the immunobiological understanding of many cancers, has directly led to this outcome. Conversely, for melanoma over the same time period, incidence rates increased while mortality rates remained unchanged [1]. Among solid tumors worldwide, the incidence rate for cutaneous melanoma is the fastest growing and three-year survival rates for patients with metastatic melanoma remains at roughly 15% [2]. The primary determinant for a melanoma patient’s long-term clinical outcome is metastasis, where spread to the draining lymph nodes alone significantly decreases patient survival [3, 4]. From the draining lymph nodes, melanoma often metastasizes to the liver, lungs, and brain leading to poor survival outcomes [5, 6].

Recently, substantial progress has been made in treating metastatic melanoma through the use of checkpoint blockade (CPB) inhibitor treatments. The discovery of two key T-cell regulators: cytotoxic T lymphocyte-associated antigen (CTLA-4) and programmed cell death protein 1 (PD-1) has led to clinical treatment options not available a decade ago. CTLA-4 inhibits activated T-cells through engagement with B7-1 and B7-2, preventing them from accessing CD28 on activated T-cells [7–11]. Using antibodies to block CTLA-4 from binding B7-1 and B7-2 frees them up to engage CD28 eliciting cytokine production and proliferation [12, 13]. Similarly, the PD-1/PD-L1 axis, a negative regulator of T-cell activation can also be targeted with antibodies [14–16]. The introduction of CPB in the treatment of melanoma is expected to reduce 5-year mortality rates [17]. However, these recent improvements in metastatic melanoma treatment are not yet curative and will have no effect on melanoma incidence rates. Common genetic variances and mutations in certain genes (reviewed in [2]) have been linked to melanoma; however, lifestyle choices remain the single largest preventable driver of disease. Individuals who are repeatedly exposed to ultraviolet radiation (i.e., individuals who work in the sun or tan for leisure) are at an increased risk for developing cutaneous melanoma. For these reasons, an effective melanoma vaccine is likely to be the most effective means to reduce incidence rates in these specific individuals.

*Listeria monocytogenes* (Lm) is a facultative intracellular bacterium, which through the use of the pore-forming toxin listeriolysin-O (LLO), escapes the phagosome to avoid lysosomal killing [18]. Once in the cytoplasm, *L. monocytogenes* is capable of replicating and gaining direct access to neighboring cells. The virulence associated genes *actA* and *plcB* facilitate spread by the polymerization of actin [19] and escape from a secondary endosome [20], respectively. Cytoplasmic replication and the less than 100% success rate of phagosomal escape means that Lm peptides can be presented by both MHC-I and MHC-II complexes, eliciting both CD8^+^ and CD4^+^ T-cell responses [21], both of which have been shown to be important in the elimination of cancer [22]. Several Lm cancer vaccination platforms have fused tumor-associated antigens (TAA) to the LLO peptide [22–24]. Due to its proinflammatory properties [25] and lack of pathogenesis in human subjects [26], we developed an *actA:plcB* double mutant with the TAA peptide fused to the first 100 amino acids of ActA [27, 28]. Fusing TAAs to ActA results in the TAA being delivered directly to the cytoplasm and thus the MHC-I processing compartment. Others have shown that compared to the LLO fusion, fusions to other Lm genes such as *inlB* can induce a greater CD8^+^ T-cell response [29] when expressed in an *actA*:*inlB* double mutant vaccine vector. Therefore, we hypothesized that delivery of TAAs directly to the cytoplasm *via* this method will improve CD8^+^ T-cell responses and thus tumor killing ability. Here, we describe an approach using a well-defined *L. monocytogenes* vector with an *actA*-TAA fusion to produce a melanoma vaccine that is effective while remaining highly attenuated.

The B16F10 cell line is the most commonly utilized mouse melanoma model and is characterized by its aggressive invasive growth and poor immunogenicity [30, 31]. We utilized a B16F10 strain expressing the immunodominant epitope of chicken ovalbumin (OVA_257-264_) [32], which in this model acts as a tumor-associated neo-antigen. This is a well-established approach to investigate cancer vaccination [33]. In this study we show that mice vaccinated with TAA-expressing Lm strains are significantly protected from cutaneous melanoma. We go on to show that when given CPB therapy following vaccination and melanoma challenge, resistance to tumor growth is enhanced compared to controls.

## RESULTS

### Vaccination with *L. monocytogenes* expressing tumor antigens elicits an epitope specific CD8 T-cell response

**Figure 1:**
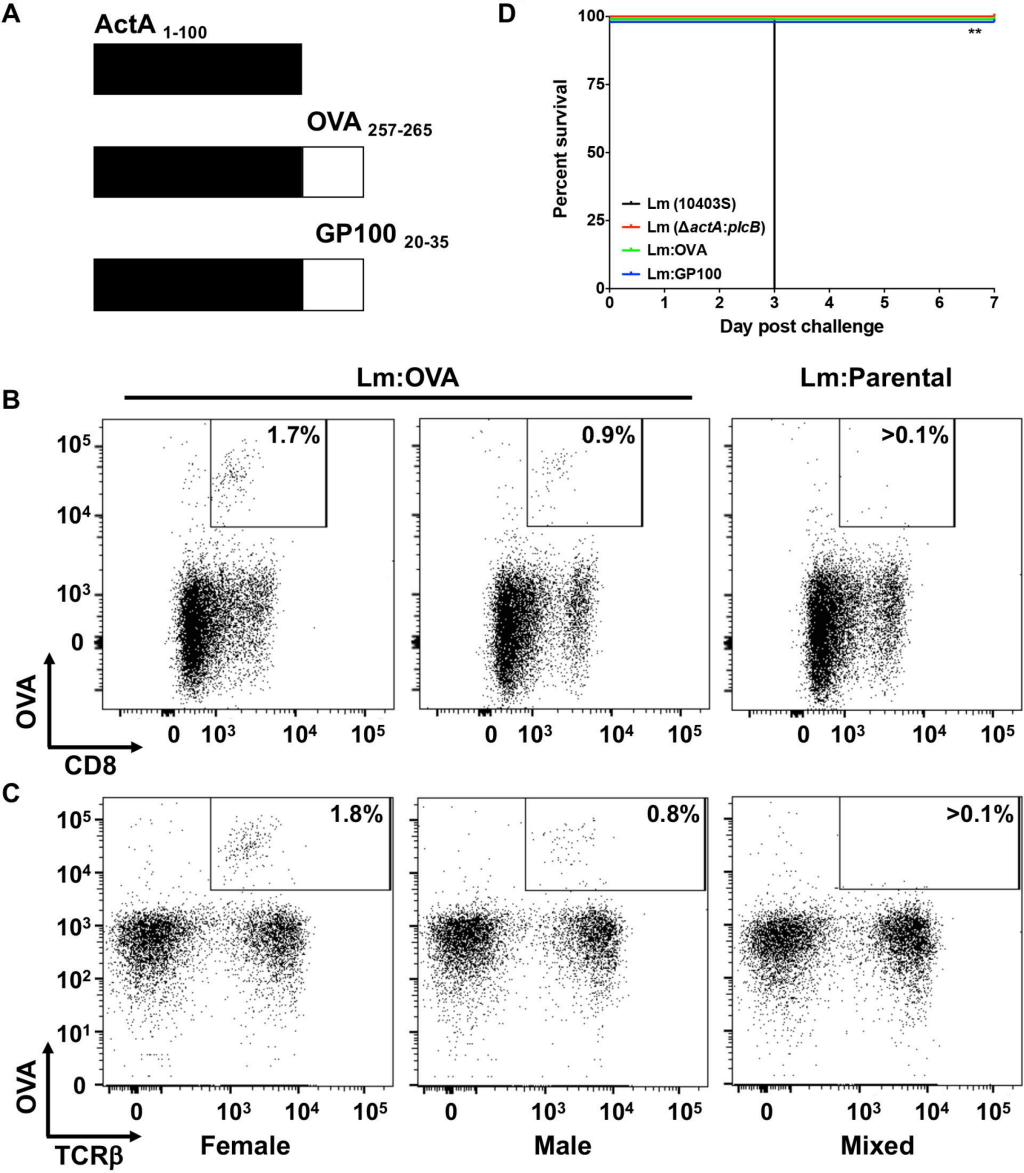
Attenuated *L. monocytogenes* expressing TAA induces specific CD8^+^ T-cell response. (**A**) Schematic of chicken ovalbumin (OVA) or mouse GP100, both tumor-associated antigens, fused to the first 100 amino acids of a truncated actin-assembly inducing protein (ActA). Along with a mutated phospholipase C (*plcB*), the introduction of the immunodominant epitope of OVA (AAs 257-265), create the OVA-expressing *L. monocytogenes* vaccine strain (Lm: OVA). Female (left panel) and male (middle panel) mice vaccinated with 2 × 10^4^ CFU of Lm: OVA develop OVA specific CD8^+^ (**B**) and TCRβ^+^ (**C**) cells compared to mice receiving the Lm: Parental vaccine (Δ*actA: plcB*), right panel. (**D**) Survival of mice (*n* = 4 per group) receiving 2 × 10^7^ CFU of either *L. monocytogenes* strain 10403S (black), Lm: Parental (red) Lm: OVA (green), or Lm: GP100 (blue). Statistical analysis was performed as a Mantel-Cox Log-rank test (*p* = 0.0074).

We previously described the *Listeria monocytogenes* strain deficient in both actin-assembly inducing protein (*actA*) and phospholipase C (*plcB*) [28], which was used as the vaccine platform in this study. This double mutant is completely attenuated in humans, lacking the ability to directly enter a neighboring cell or escape a secondary membrane [34], and importantly it is unable to cause neuro-invasiveness [35]. The immuno-dominant peptide sequence (amino acids 257-264) from chicken ovalbumin (OVA) were expressed as fusions to the first 100 amino acids of ActA (Figure 1A). Female and male C57BL/6 mice vaccinated with 2 × 10^4^ CFU of the OVA-expressing *L. monocytogenes* strain developed a robust OVA specific CD8^+^/TCRβ^+^ T-cell response while those vaccinated with the parental *L. monocytogenes* strain did not (Figure 1B and 1C). As previously reported by us and others [28, 29], a single vaccination with increasing doses of Lm: OVA (2 × 10^5^ or 2 × 10^6^ CFU) vaccine failed to significantly increase the numbers of OVA specific CD8^+^/TCRβ^+^ T-cells (Supplementary Figure 1A and 1B). Importantly, the Δ*actA*:*plcB* (Lm: Parental) as well as the *actA*-OVA fusion (Lm: OVA) vaccine strains were completely attenuated with i. v. vaccination doses up to 2 × 10^7^ CFU (Figure 1D). In contrast, the virulent *L. monocytogenes* from which the vaccine strain was generated, *L. monocytogenes* strain 10403S, produced 100% mortality by day 3 post challenge (Figure 1D). Furthermore, neither the Lm: Parental or Lm: OVA vaccine strains elicited significant morbidity, as measured by weight change, whereas the virulent strain did (Supplementary Figure 1C).

**Figure 2:**
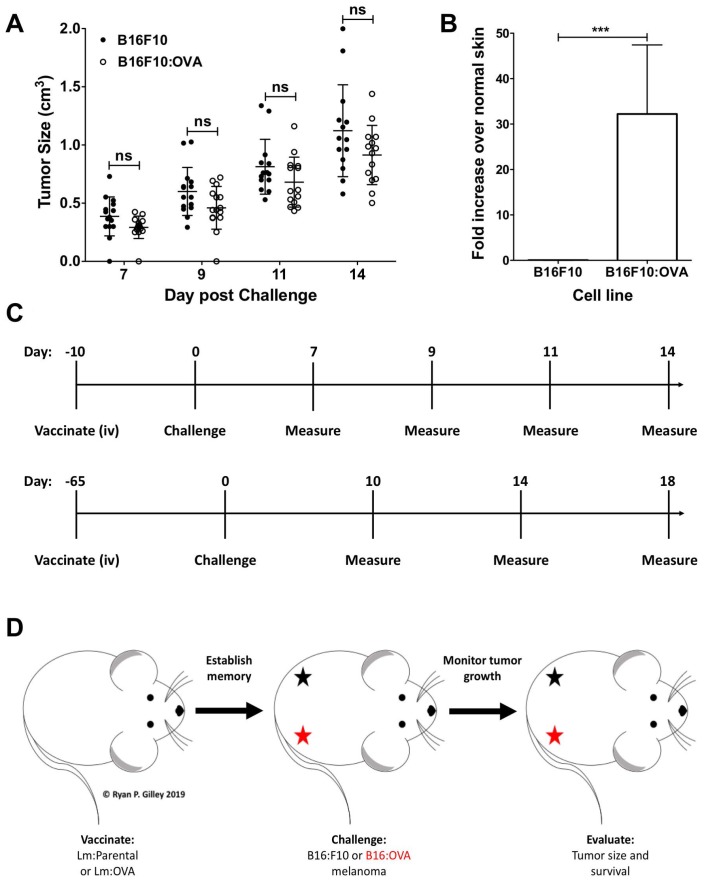
B16F10: OVA cells grow *in vivo* and continue to express OVA. (**A**) When injected, B16F10 and B16F10: OVA melanoma cells grow on the hindquarters of unvaccinated female mice at similar rates (*n* = 15 per group); two-way ANOVA with Bonferroni post-test. (**B**) qRT-PCR from resected tumor RNA from unvaccinated female mice (*n* = 3 per group) showing B16F10: OVA cells expressing OVA *in vivo*; two-tailed paired *t*-test (*p* = 0.0002). (**C**) Schematic showing vaccination, challenge, and tumor measurement schedule for a primary (10 days post vaccination challenge, upper panel) and memory recall (65 days post vaccination challenge, lower panel). (**D**) Mouse challenge schematic showing injection site of B16F10 (black star) and B16F10: OVA (red star) on the hindquarters of mice.

We utilized the well characterized B16F10 mouse melanoma cell line derived from C57BL/6 mice [30, 31] as well as an isogenic OVA-expressing B16F10 cell line (B16: OVA), both of which readily grew in unvaccinated mice (Figure 2A). Critically, the B16: OVA cell line expresses the TAA OVA *in vivo* while the parental B16F10 does not (Figure 2B). To test the vaccine’s ability to elicit a primary (10 days post vaccination) or memory (65 days post vaccination) response, mice were vaccinated intravenously (i. v.) and then challenged with melanoma and evaluated as shown in Figure 2C. The experiments were internally controlled with mice being vaccinated with a single Lm strain but challenged with both B16F10 cell lines, then monitored (Figure 2D).

### Mice vaccinated with Lm expressing TAA are protected from melanoma

**Figure 3:**
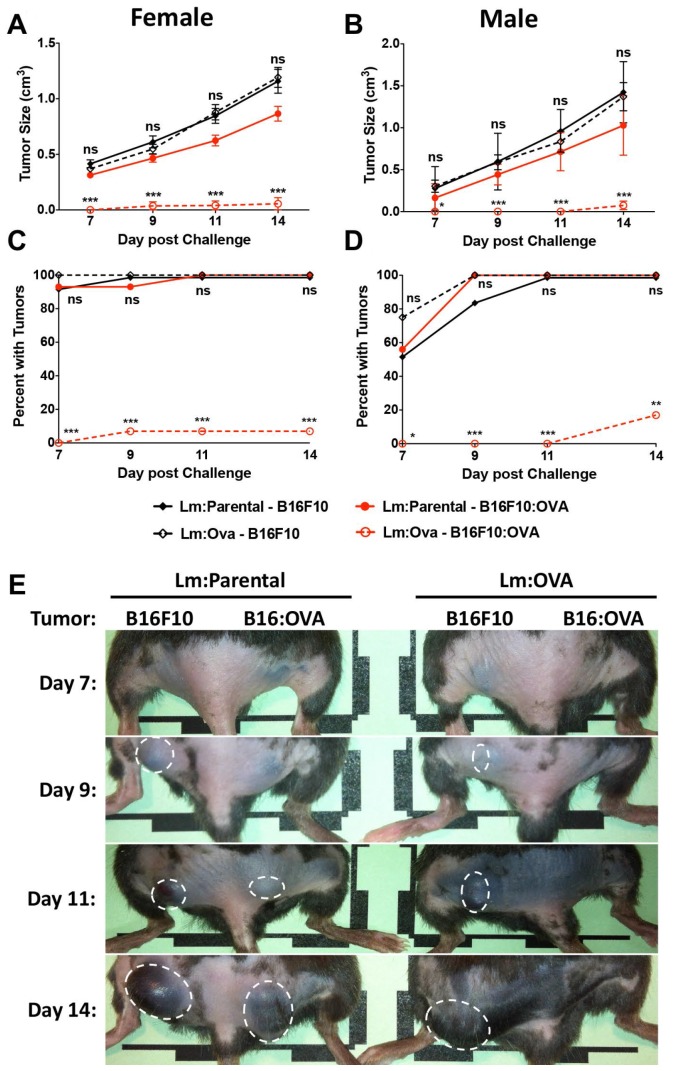
Lm: OVA vaccination significantly protects mice from B16F10: OVA during primary challenge. B16F10: OVA tumor sizes are significantly smaller in Lm: OVA vaccinated mice (open red) than they are in Lm: Parental vaccinated mice (closed red) in both (**A**) female (*n* = 15 per group) and (**B**) male mice (*n* = 8–13 per group). Conversely, B16F10 tumor sizes were not different in Lm: OVA vaccinated mice (open black) compared to Lm: Parental vaccinated mice (closed black). The number of B16F10: OVA tumors in (**C**) female and (**D**) male mice were significantly less numerous in Lm: OVA vs. Lm: Parental vaccinated mice; B16F10 tumor numbers were not significantly different numerous in Lm: OVA vs. Lm: Parental vaccinated mice. (**E**) Representative tumor images of female mice receiving either the Lm: Parental or Lm: OVA vaccine and challenged with both B16F10 and B16F10: OVA melanoma cells. Tumor sizes and numbers were analyzed by two-way ANOVA with Bonferroni post-test. ^*^ = *p* ≤ 0.05, ^**^ = *p* ≤ 0.01, ^***^ = *p* ≤ 0.001.

**Figure 4:**
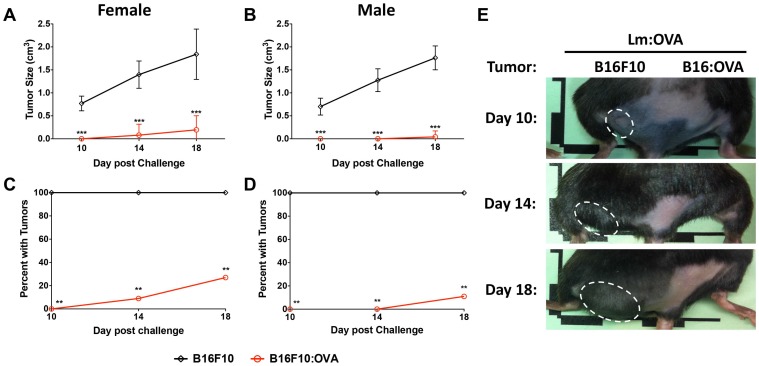
Memory responses in Lm: OVA vaccinated mice are protective. B16F10: OVA tumors (red line) in Lm: OVA vaccinated (**A**) female and (**B**) male mice were significantly smaller than B16F10 tumors (black line) in those same mice. The number of B16F10: OVA injection sites with tumors were significantly less numerous than at B16F10 injection sites 65 days post Lm: OVA vaccination in (**C**) female and (**D**) male mice. (**E**) Representative tumor images of female mice vaccinated with Lm: OVA and injected with both B16F10 and B16F10: OVA. Tumor sizes and numbers were analyzed by two-way ANOVA with Bonferroni post-test. ^*^ = *p* ≤ 0.05, ^**^ = *p* ≤ 0.01, ^***^ = *p* ≤ 0.001; *n* = 9–11 per group.

Mice were vaccinated with 2 × 10^4^ CFU of either the control vector (Lm: Parental) or Lm: OVA. Mice were then challenged 10 dpv with 2 × 10^5^ cells of both B16F10 lines on either rear flank as shown (Figure 2D). B16F10 cells expressing OVA were significantly restricted in their growth in mice receiving the Lm: OVA vaccine (Figure 3A–3E). In agreement with the analyses of vaccine-elicited T-cell responses in Supplementary Figure 1A and 1B, mice receiving a 100-fold higher vaccination dose (2 × 10^6^ Lm: OVA), were not any more protected from cutaneous tumors than those receiving 2 × 10^4^ (Supplementary Figure 1D). The effectiveness of vaccination to TAA can be limited by tolerance but in this preclinical model, OVA is acting as a neo-antigen and induces a robust immune response. Tolerance can be overcome by adjuvants or the use of microbial vectors. To evaluate protection when mice were vaccinated with an endogenous TAA, the immunodominant GP100 peptide was fused to ActA and mice were vaccinated and challenged with B16F10 as described above. The Lm vaccine platform expressing an endogenous melanoma-associated antigen, GP100, showed similar effectiveness as the Lm: OVA vaccine (Supplementary Figure 2). Similar to mice challenged 10 dpv, the Lm: OVA vaccine provided significant protection from B16: OVA when challenged at 65-dpv. While the B16F10 cell line formed tumors, the B16: OVA cell line failed to consistently elicit tumors in vaccinated mice (Figure 4A–4E). Although others have reported a modest decrease in tumor growth with vector alone [33], we did not notice any difference in growth kinetics of B16F10 lines between vector-vaccinated and unvaccinated mice (Figures 3A, 3B, 4A, 4B). Altogether, these data suggest that Lm: OVA induces both antigen-specific primary immunity against melanoma and a protective memory response.

### CD8^+^ T-cells are responsible for anti-tumor immunity

**Figure 5:**
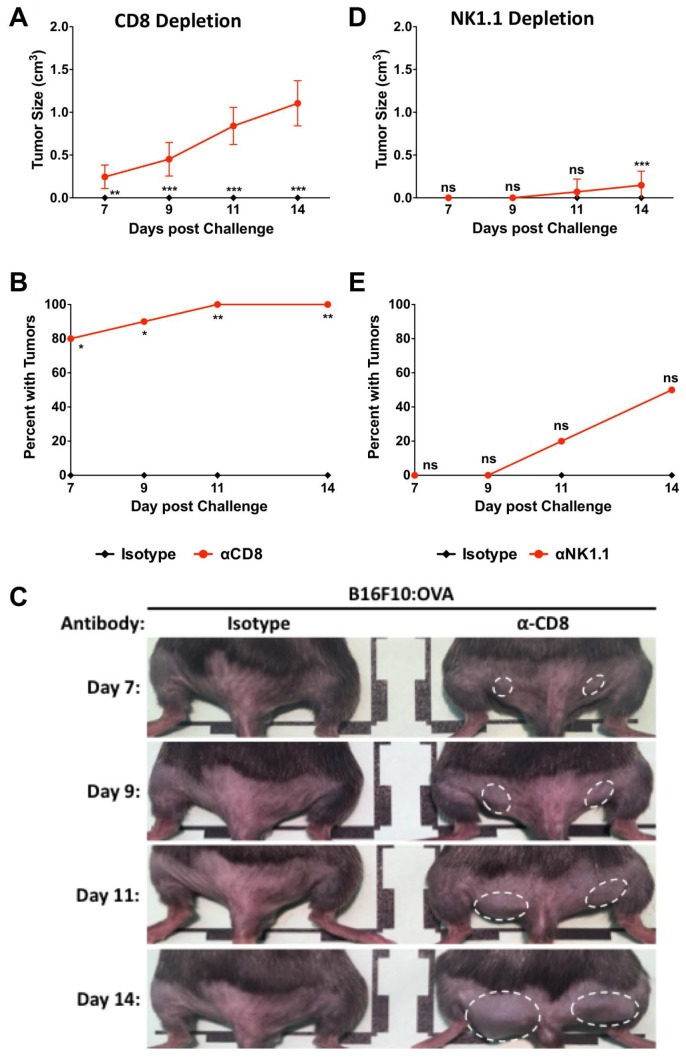
CD8^+^ cells are primarily responsible for protection at 10 days post Lm: OVA vaccination. (**A**) Tumor size, (**B**) breakthrough percentage, and (**C**) representative images from Lm: OVA vaccinated female mice depleted of CD8^+^ cells. (**D**) Tumor size and (**E**) tumor injection site breakthrough in Lm: OVA vaccinated mice depleted of NK1.1^+^ cells. All mice were vaccinated with Lm: OVA and challenged with B16F10: OVA melanoma cells. Tumor sizes and numbers were analyzed by two-way ANOVA with Bonferroni post-test. ^*^ = *p* ≤ 0.05, ^**^ = *p* ≤ 0.01, ^***^ = *p* ≤ 0.001; *n* = 10 per group.

We previously showed that the Lm: OVA vaccine induces a robust antigen-specific CD8^+^/TCRβ^+^ T-cell response (Figure 1B and 1C) and [28]. However, both CD8^+^ T-cells and NK cells have been shown to be important in Lm vaccine-mediated immunity [36]. To test if CD8^+^ T-cells or NK cells were required for vaccine mediated immunity following Lm: OVA vaccination, these cells were separately depleted with antibodies. Two days prior to B16F10: OVA challenge (which was eight dpv) and then every four days thereafter, Lm: OVA vaccinated mice received an i. p. injection of CD8^+^ or NK cell depleting antibody (Supplementary Figure 3A–3C). Ten days after vaccination (two days post depletion), mice were challenged intradermally as described above and tumor formation was monitored for 14 days. Depletion of these cell types suggests that CD8^+^ T-cells are necessary for primary anti-tumor immunity (Figure 5A–5C). Although NK cells have been directly linked *via* type I IFN to the anti-tumor properties derived from a recombinant *L. monocytogenes* vaccine [37], they do not play a significant role in Lm: OVA derived anti-tumor immunity in this study (Figure 5D, 5E and Supplementary Figure 3D).

**Figure 6:**
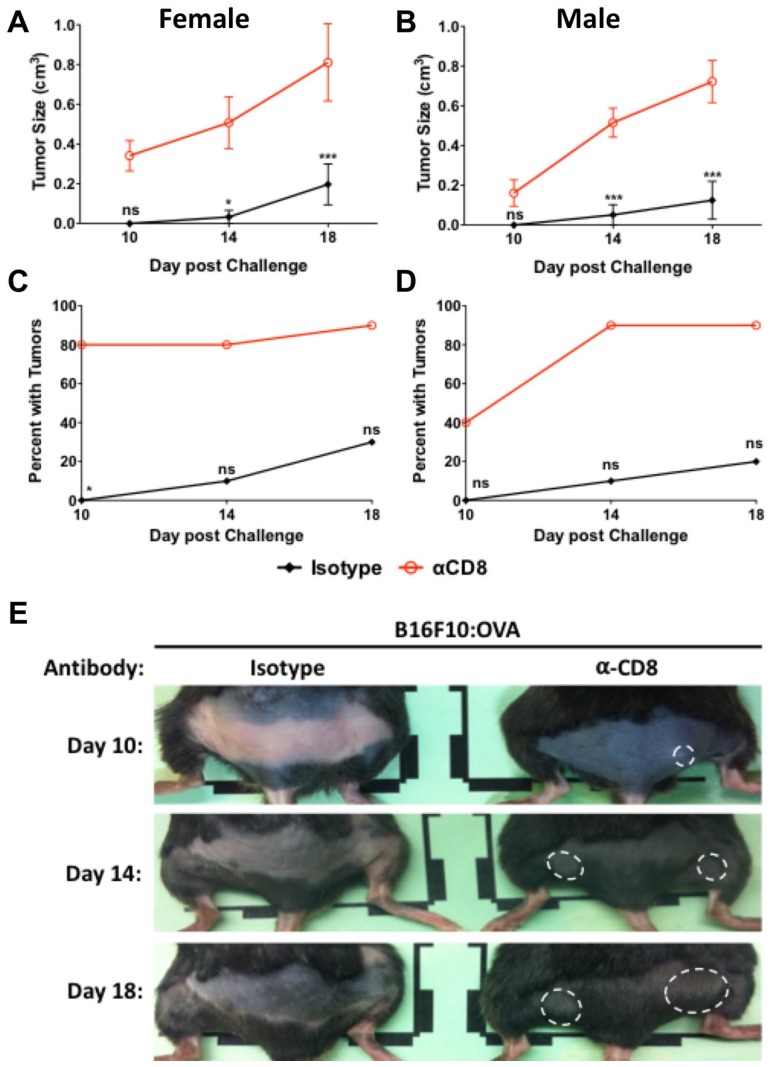
CD8^+^ cells provide protection 65 days after vaccination. B16F10: OVA tumor sizes in (**A**) female and (**B**) male mice vaccinated with Lm: OVA and depleted of CD8^+^ cells were significantly larger at 65 days post vaccination than mice receiving isotype control. Injection site tumor breakthrough percentage in Lm: OVA vaccinated (**C**) female and (**D**) male mice receiving either isotype control or α-CD8 antibody. (**E**) Representative images of female mice vaccinated with Lm: OVA, depleted of CD8^+^ cells, and challenged with B16F10: OVA melanoma cells. Tumor sizes and numbers were analyzed by two-way ANOVA with Bonferroni post-test. ^*^ = *p* ≤ 0.05, ^**^ = *p* ≤ 0.01, ^***^ = *p* ≤ 0.001; *n* = 10 per group.

To evaluate cellular protection in animals with established memory, mice were vaccinated with Lm: OVA, depleted, and challenged as described in the 10-dpv depletion model except analyzed on the 65-dpv timeline as shown in (Figure 2C). Those mice receiving αCD8 injections developed larger and more numerous tumors than those receiving control antibodies (Figure 6A–6E). The totality of these results suggests that TAA-specific CD8^+^ T-cells are predominantly responsible for both the primary and recall protection.

### Checkpoint blockade treatment improves outcomes

While most vaccinated animals in this study did not develop tumors, the stringent melanoma challenge utilized in these studies can result in up to 30% of vaccinated animals ultimately developing tumors. It was also noted that the percentage of vaccinated animals that developed tumors increased as a function of time from tumor challenge. We hypothesized that these observations could be due to immune tolerance through engagement of immune checkpoint pathways. To test if checkpoint blockade could further improve vaccine-mediated anti-tumor immunity, we tested the effectiveness of combining checkpoint blockade with Lm-based vaccination. Unvaccinated mice treated with either αPD-1, αPD-L1, or αCTLA-4 checkpoint blockade inhibitor antibodies experienced a modest yet not significant decrease in tumor volume and an increase in mean time to death compared to control animals (Supplementary Figure 4A and 4B).

**Figure 7:**
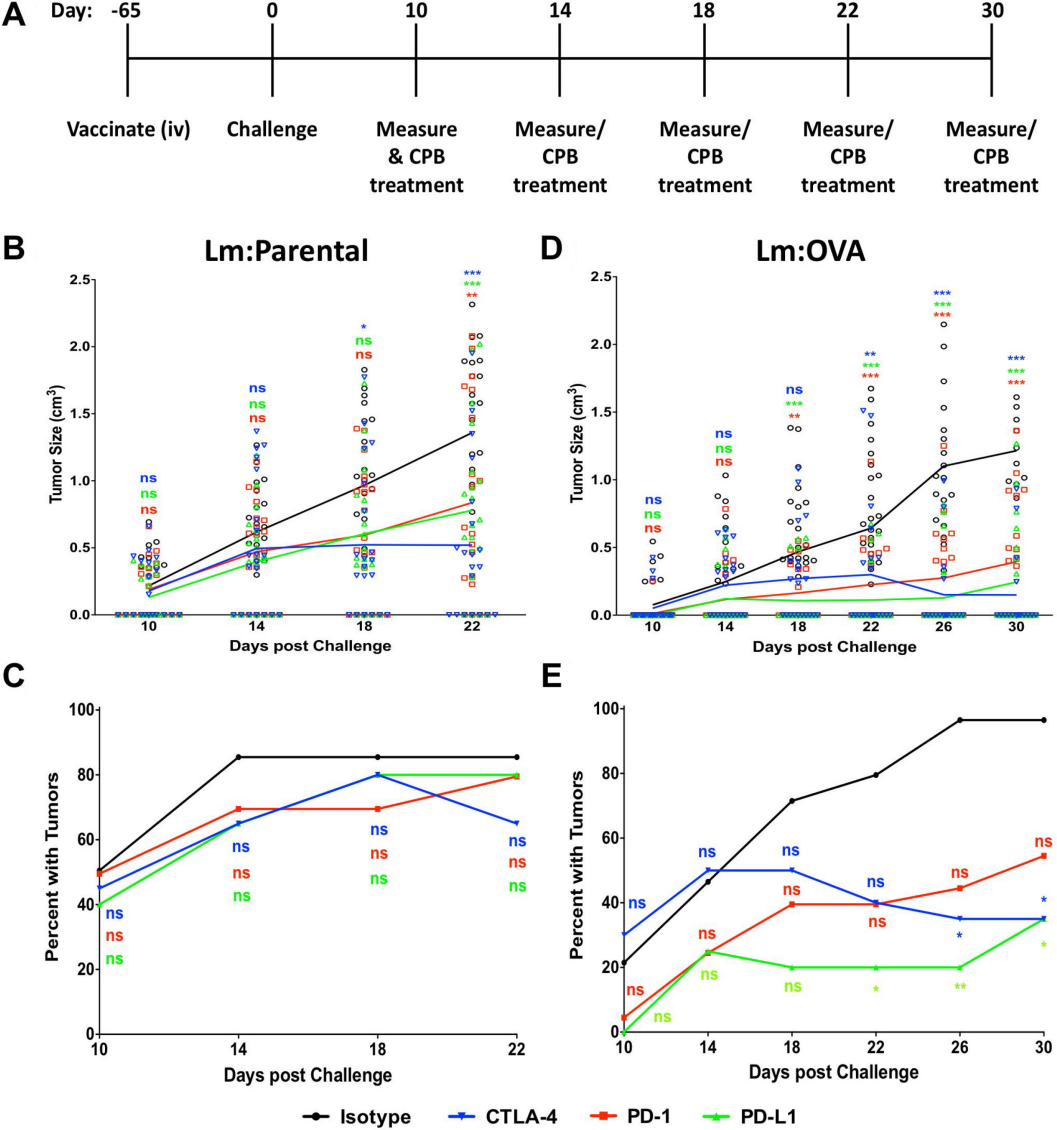
Checkpoint blockade treatment enhances Lm: OVA vaccination tumor prevention. (**A**) Schematic of vaccination, checkpoint blockade treatment, and tumor measurement schedule. Lm: Parental (*n* = 20 per group) vaccinated experiments ended at 22 days post challenge while Lm: OVA (*n* = 20–28 per group) vaccinated experiments ended at 30 days post challenge. (**B**) Tumor size and (**C**) injection site tumor breakthrough percentage in Lm: Parental vaccinated mice challenged with B16F10: OVA melanoma cells. (**D**) Tumor size and (**E**) injection site tumor breakthrough percentage in Lm: OVA vaccinated mice challenged with B16F10: OVA melanoma cells. Following B16F10: OVA challenge, all mice were given either isotype (black), α-CTLA-4 (blue), α-PD-1 (red), or α-PD-L1 (green). Tumor sizes and numbers were analyzed by two-way ANOVA with Bonferroni post-test. ^*^ = *p* ≤ 0.05, ^**^ = *p* ≤ 0.01, ^***^ = *p* ≤ 0.001.

To evaluate the ability of checkpoint blockade inhibitor treatment to enhance vaccine mediated immunity, mice were vaccinated and then treated with checkpoint blockade antibodies or control antibodies according to the schematic in Figure 7A. Mice given the Lm: Parental strain and then either αPD-1, αPD-L1, or αCTLA-4 every four days starting at 10-days post challenge (dpc) had decreased tumor size and a modest yet not significant reduction in tumor number (Figure 7B and 7C). In contrast, mice vaccinated with Lm: OVA and receiving checkpoint blockade treatment had a significant decrease in tumor size and number compared to those mice receiving control treatments (Figure 7D and 7E). These data suggest that check point blockade treatment bolsters vaccine-mediated anti-tumor immunity in a mouse model of cutaneous melanoma.

## DISCUSSION

*L. monocytogenes* has long been a desirable vector choice for attempting to harness the immunogenic properties of pathogens for treating numerous human cancers [38, 39]. Despite some inherent challenges with use of live-attenuated bacteria as a vaccine platform, including safety concerns and adverse reactions to the bacteria [40], *L. monocytogenes* based vectors for cancer vaccines have numerous advantages. It is a well-characterized model organism with well-defined virulence determinants encoded within a 10-kb region of the chromosome called the PrfA regulon [41]. As a normally pathogenic bacterium, *L. monocytogenes* contains PAMPS with adjuvant properties that induce inflammatory responses through activation of TLR 1 (peptidoglycan), TLR 2 (lipoteichoic acid) [42, 43] and TLR 5 (flagellin) [44]. As reported by others, vaccination with attenuated recombinant Lm induces IL-1, -2, -6, -12, Type I IFN and TNFβ [33]. Unlike viral vectors, bacterial vector systems also have the advantage of not being tightly constrained by the size of the recombinant tumor antigen (s) allowing for the expression of large recombinant antigen (s). This would presumably allow for the insertion of multiple TAAs, an approach that will likely be necessary for the practical translation of a preventative or therapeutic melanoma vaccine.

Although others have reported similar protection with oral or intra-muscular routes of vaccination [29], the safety of this specific strain [26, 28] and newer phase I safety trials [45] suggest vaccination *via* the i.v. route is not a barrier to utilization. Additional phase I & II clinical trials aimed at evaluating safety and effectiveness in treating various human cancers using recombinant *L. monocytogenes* vectors have proven to be both safe and effective with additional Lm-based vaccines in preclinical development [46, 47]. Likewise, other live attenuated bacterial species have been successfully used to treat cancer, best exemplified by the *Mycobacterium bovis* BCG strain for the treatment of bladder cancer [48].

We report that a single low dose (2 × 10^4^ CFU) of *L. monocytogenes* expressing a TAA was sufficient to elicit a protective CD8^+^ T-cell response that protected both male and female mice even at extended challenge times, i.e., 65-dpv. In addition to testing our vaccine at 65-dpv, we also saw protection, although somewhat diminished, at 82-dpv from a single vaccination (Supplementary Figure 4C). This suggests the Lm vector is capable of inducing T-cell memory that would support long-term immunity. Because OVA is highly immunogenic, we used a preclinical mouse model of cutaneous melanoma that included a stringent tumor challenge designed to elicit a tumor at each challenge site. Encouragingly, even 65-days after a single low-dose vaccination, significant protection against tumor challenge was observed suggesting significant potential as a preventative vaccine.

Elimination of tumor cells within an organism can be challenging and vaccination against self-antigens is a difficult task even when those antigens are associated with a tumor. There are numerous reasons for this including tolerance mechanisms and possibly a lack of effective MHC-I presentation. Although no definitive link has been made to immune evasion, human melanoma is associated with poor MHC-I presentation [49]. Along these lines, the B16F10 cell line has a rather poor expression of MHC-I [50, 51]. Crucially, Lm infection elicits IFN-γ, IL-12, and TNFα, cytokines associated with increased antigen processing and presentation, however the direct immunological utility of this in the described model was not specifically tested.

Since immunity *via* this vaccine platform is mediated through T-cells, we tested whether blocking the T-cell regulators CTLA-4 and PD-1 would improve disease outcomes. Not surprisingly, blockade of both interactions: CTLA-4/B7-1 and B7-2 with anti-CTLA-4 antibody, or PD-1/PD-L1 with either anti-PD-1 or anti-PD-L1 antibody resulted in decreased tumor size in unimmunized mice. Application of CPB inhibitor antibody in vaccinated mice resulted in a substantial divergence from vaccinated mice receiving isotype control. However, the most striking difference between vaccinated and unvaccinated mice receiving CPB treatment is the percentage of tumors in vaccinated mice that failed to develop. Although CPB did have positive effects on unvaccinated mice, similarly to clinical observations with anti-CTLA-4, anti-PD-1, and anti-PD-L1 treatment, these results suggest that when pre-existing immunity is unlocked by blocking immune-suppressive interactions, outcomes may be significantly improved. It is worth noting *L. monocytogenes* infection upregulates PD-L1 expression. As such, Mkrtichyan *et al*. found that addition of anti-PD-L1 antibody significantly improved outcomes in a TC-1 mouse tumor model with a Lm-LLO-E7 therapeutic vaccination [52]. Data presented in the current study is consistent with those of Mkrtichyan, and together these data suggest that CPB can improve outcomes from Lm-based preventative and therapeutic cancer vaccines.

These results contrast with a human trial comparing GP100 peptide vaccination and CTLA-4 immunotherapy where there was no added benefits to vaccination and immunotherapy compared to immunotherapy alone [53]. Whether the anti-tumor effects of the GP100-expressing *L. monocytogenes* strain is enhanced by CPB is the focus of ongoing investigations. While there are numerous reasons why the current study could show a synergistic benefit to vaccination and CPB treatment, the properties of the vaccine vector likely influence outcomes. Which measurable differences are vector specific rather than TAA specific requires additional investigation.

Altogether, data presented in this study demonstrate that expression of TAA as an *actA*-fusion from recombinant Lm induces significant anti-tumor immunity as a preventative melanoma vaccine, and that immunity can be enhanced by treatment with CPB therapy after vaccination. Although our data provides substantial evidence that Lm-based vaccines are efficient preventative vaccines, we did not evaluate the potential of this vaccine under therapeutic conditions. Therapeutic cancer vaccines have been elusive, but the properties of Lm that make it a good preventative vaccine could also be beneficial as a therapeutic vaccine. This is especially true of the ability of recombinant Lm to induce MHC-I and MHC-II dependent responses, the ability to express large recombinant antigens, and the ability to induce pro-inflammatory cytokines. Utilization of personalized medicine approaches like neo-antigen-based therapeutic vaccines for melanoma have shown great promise [54, 55]. Along these lines, expression of OVA within the tumor acts similarly to a neo-antigen since it is found nowhere else in the mice. This makes the possibility of delivering personalized, neo-antigens in a therapeutic manner while retaining efficaciousness a strong possibility. Despite not being directly tested in this study, Lm-based vaccination platforms such as the one described here is a well-suited vaccine platform for delivery of a neo-antigen based or other approaches to cancer vaccination.

## MATERIALS AND METHODS

### Mouse strains

All mice used in this study were C57BL/6 mice purchased from Jackson Labs (Bar Harbor, ME, USA) or bred in the UTHSCSA animal facility.

### Bacterial strains

*Listeria monocytogenes* strain 10403S double mutant with a truncated actin-assembly inducing protein precursor (*actA*) and phospholipase C (*plcB*) served as the parental vaccine strain (Lm: Parental) in all experiments and has been previously described [28]. To this parental mutant strain, the immunodominant epitope, amino acids 257–264, of chicken ovalbumin (OVA) was fused to *actA* as described to create an OVA-expressing *L. monocytogenes* strain (Lm: OVA) [28]. The GP100-expressing *L. monocytogenes* strain was created by inserting amino acids 20-35 from mouse GP100 into the pLP2 plasmid fused to ActA *via* restriction digestion and inserted on the chromosome in-frame in the 10403S double mutant as previously described [27, 56]. *L. monocytogenes* strains were grown in Brain Heart Infusion broth (BD, Franklin Lakes, NJ, USA) to an OD_600_ of 0.600. Bacteria were then pelleted at 4000×g for 10 minutes and washed in sterile phosphate buffered saline (PBS), resuspended cells were then serially diluted in PBS to desired concentration for vaccinations. Mice were vaccinated intravenously with 2 × 10^4^ CFU of either Lm: Parental or Lm: OVA in 100 μL of PBS using a 30-guage needle. Inoculum counts were confirmed by plating serial dilutions and extrapolation of CFU.

### Collection and assessment of splenocytes and livers

Organs from vaccinated mice were collected 10 days post vaccination and placed on ice in DMEM supplemented with 10% FBS. Harvested spleens were forced through a 70 μm nylon filter (Fisher, Hampton, NH, USA), which was then rinsed three times with collection media and pelleted at 800×g for 5 min. Supernatant was decanted and pellet was resuspended in collection media and red blood cell lysis buffer (Sigma, St. Louis, MO, USA) for 1 min at room temperature. Collection tubes were then filled with 50 mL of FACS wash buffer (0.05% BSA in PBS) and spun as before, cells were then resuspended in FACS staining buffer (1% BSA in PBS) and enumerated. A total of 1 × 10^6^ cells in 50 μL were stained with either MHC Tetramer, H-2 Kb OVA (MBL: T03000); TCRβ clone H57-597 (BD Pharmingen: 553174); CD8α clone 53-6.7 (BD Pharmingen: 553030 or BD Pharmingen: 553033) for depletion analysis, all at 1:500 in FACS tubes for 30 min on ice. After 30 minutes, FACS tubes were filled with FACS wash buffer and spun as before. Supernatant was then decanted and cells were resuspended in 10% neutral buffered formalin and analyzed on a BD LSR-II (Franklin Lakes, NJ, USA) at the UTHSCSA flow cytometry core.

### Cell lines

The mouse melanoma cell line B16F10 has been previously described [31]. Along with the B16F10 melanoma line, we utilized a B16F10 strain expressing chicken ovalbumin (B16: OVA) [57]; both B16F10 cell lines were kindly provided by Tyler J. Curiel (UTHSCSA). Both were grown in Dulbecco’s Modified Eagle Medium (Sigma) supplemented with 10% FBS (HyClone, Thermo, Logan, UT, USA), 100 U/mL penicillin, and 100 μg/mL streptomycin (Thermo, Waltham, MA, USA), as per manufacturer’s recommendations (ATCC, Manassas VA, USA). For mouse challenges, cells were grown to 70–80% density, trypsinized, pelleted at 130 × g for 10 minutes, then resuspended in growth media and passed through a 70 μm nylon filter and enumerated on a hemocytometer.

### Tumor challenges and measurements

Three days prior to challenge, the fur on the hindquarters of mice was shaved. Inoculums of 100 μL were injected intradermally with a 27-gauge needle into the hindquarters of mice anesthetized with 3% isoflurane (Vet One, Boise, ID, USA). Tumors were measured from the rear at the same angle each time at predetermined times using a digital metric caliper (VWR, Radnor, PA, USA). Mice with tumors that exceeded 2 cm, bled, ulcerated, caused immobility of a limb, or some other significant impairment were euthanized; otherwise mice were euthanized at predetermined experimental endpoints using CO_2_ asphyxiation and confirmed with cervical dislocation.

### qRT-PCR analysis

B16F10 and B16: OVA tumors were grown on unvaccinated mice as described above; at ten days post challenge, tumors were resected and homogenized in trizol. RNA was extracted and used to make cDNA, from which levels of OVA transcript (4331182; Gg03366807) was determined *via* Taqman qRT-PCR with mouse GAPDH (4351370; Mm99999915) serving as the internal control. Levels of OVA in each tumor was determined as fold change compared to normal mouse skin collected from the hindquarters of non-challenged mice (*n* = 3).

### Checkpoint blockade and cell depletion

Mice were given intraperitoneal injections of 200 μg of each respective antibody (as outlined in each specific experiment) diluted to a total of 100 μL in sterile PBS using a 27-guage needle. Checkpoint blockade inhibitor antibodies were purchased from BioXCell (West Lebanon, NH, USA); αCTLA-4 clone 9H10 (BE0131), αPD-1 clone 29F.1A12 (BE0273), αPD-L1 clone 10F.9G2 (BE0101), or Isotype clone 2A3 (BE0089). CD8 depletion was performed with the rat anti-mouse αCD8 clone 2.43 and isotype clone 2A3. NK depletion was performed with αNK1.1 clone PK136 (BE0036) and isotype clone C1.18.4 (BE0085), both from BioXCell.

### Statistical analyses

All data sets and statistical analyses were performed using Graphpad Prisim 5 software (Graphpad, La Jolla, CA, USA).

### Ethics statement

All mouse experiments were reviewed and approved by the Institutional Animal Care and Use Committees at The University of Texas Health San Antonio (Protocol # 20170091AR). Animal care and experimental protocols adhered to Public Law 89-544 (Animal Welfare Act) and its amendments, Public Health Services guidelines, and the Guide for the care and use of Laboratory Animals (U.S. Department of Health & Human Services).

## SUPPLEMENTARY MATERIALS



## Abbreviations

CPB: checkpoint blockade
CTLA-4: cytotoxic T lymphocyte-associated antigen
PD-1: programmed cell death protein 1
PD-L1: programmed cell death protein ligand 1
Lm: *Listeria monocytogenes*
Lm:OVA: OVA-expressing *L. monocytogenes*
LLO: listeriolysin-O
TAA: tumor-associated antigen
ActA: actin-assembly inducing protein
PlcB: phospholipase C; i.v.: intravenous
OVA: chicken ovalbumin
dpv: days post vaccination
dpc: days post challenge; i.p.: intraperitoneal
B16F10:OVA: OVA-expressing B16F10 line
