# Supplementary material for: Checkpoint blockade inhibitors enhances the effectiveness of a Listeria monocytogenes-based melanoma vaccine
Source: Oncotarget. 2020 Feb 18;11(7):740–54. doi: 10.18632/oncotarget.27490 (PMC7041938; doi:10.18632/oncotarget.27490)
Supplement: Supplementary file 1 [file oncotarget-11-740-s001.pdf]

Checkpoint blockade inhibitors enhances the effectiveness of a Listeria monocytogenes-based melanoma vaccine

SUPPLEMENTARY MATERIALS

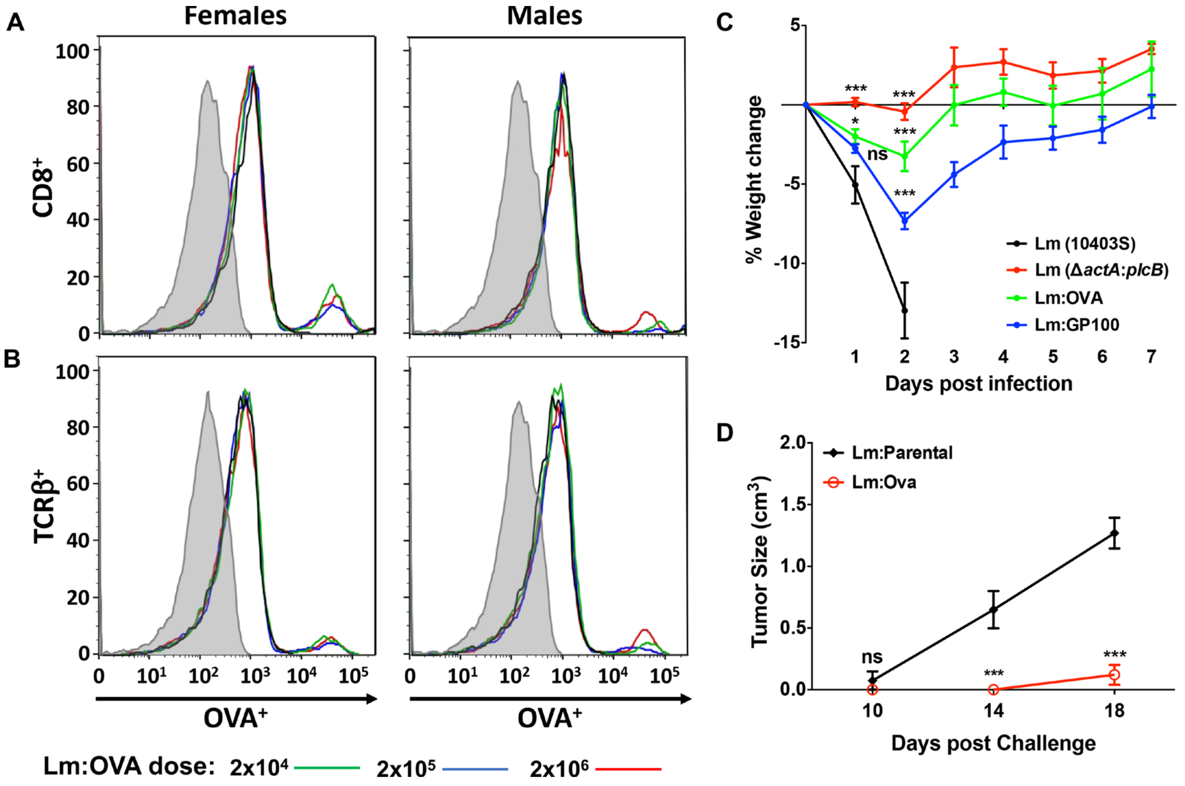

Supplementary Figure 1: Increased Lm: OVA vaccination dose does not increase immunity. OVA tetramer positive staining from (**A**) $CD8^+$ gated cells or (**B**) $TCR\beta^+$ gated cells in female (left panels) and male (right panels) mice receiving either 2×10^4 (green), 2×10^5 (blue), or 2×10^6 (red) CFU of the Lm: OVA vaccine. (**C**) Weight change in female mice ($n = 4$ per group) receiving 2×10^7 CFU i. v. of virulent *L. monocytogenes* strain 10403S (black), Lm:Δ*actA: plcB* double mutant (Lm: Parental; red), Lm: OVA (green), or Lm: GP100 (blue); weight change analysis compared to Lm:10403S. (**D**) Tumor size from female mice ($n = 4$ per group) receiving 2×10^6 CFU of either Lm: Parental (black) or Lm: OVA (red) and challenged with 2×10^5 B16F10: OVA melanoma cells 10 days post vaccination. Weight change and tumor sizes were analyzed by two-way ANOVA with Bonferroni post-test. $^*p \leq 0.05$, $^{**}p \leq 0.01$, $^{***}p \leq 0.001$.

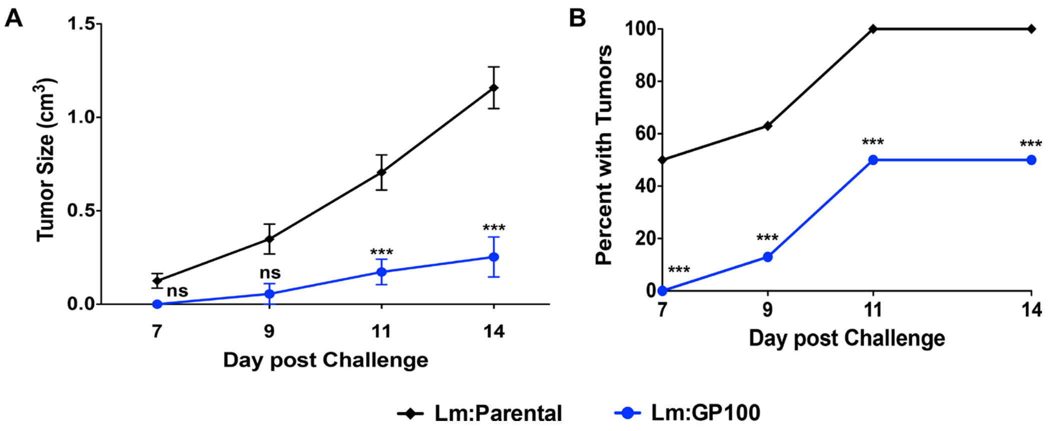

Supplementary Figure 2: Primary challenge following vaccination with the endogenous TAA GP100. (**A**) Tumor size and (**B**) breakthrough percentage of mice challenged with 2×10^5 B16F10 melanoma cells following Lm: Parental (black; $n = 8$) or Lm: GP100 (blue; $n = 4$) vaccination. Tumor sizes and numbers were analyzed by two-way ANOVA with Bonferroni post-test. $^{*}p \leq 0.05$, $^{**}p \leq 0.01$, $^{***}p \leq 0.001$.

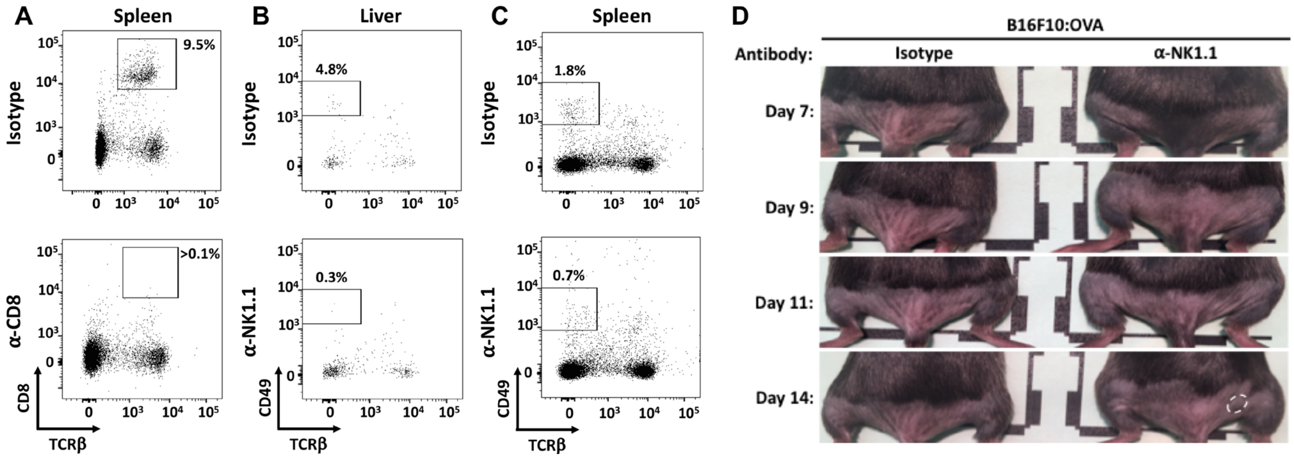

Supplementary Figure 3: Depletion of CD8 and NK cells. (**A**) Representative plot showing a 99% decrease in $CD8^+/TCR\beta^+$ cells from the spleen of female mice receiving α-CD8 antibody compared to isotype control. Representative plots showing a (**B**) 94% decrease in $CD45^+$ (gated), $CD49^+/TCR\beta^-$ NK cells in the liver and (**C**) a 61% decrease in the spleen of a female mouse receiving α-NK1.1 antibody compared to isotype control. (**D**) Representative tumor (white dashed circle) images of Lm: OVA vaccinated mice given either isotype or α-NK1.1 antibody and challenged with B16F10: OVA melanoma cells 10 days post vaccination; $n = 10$ per group.

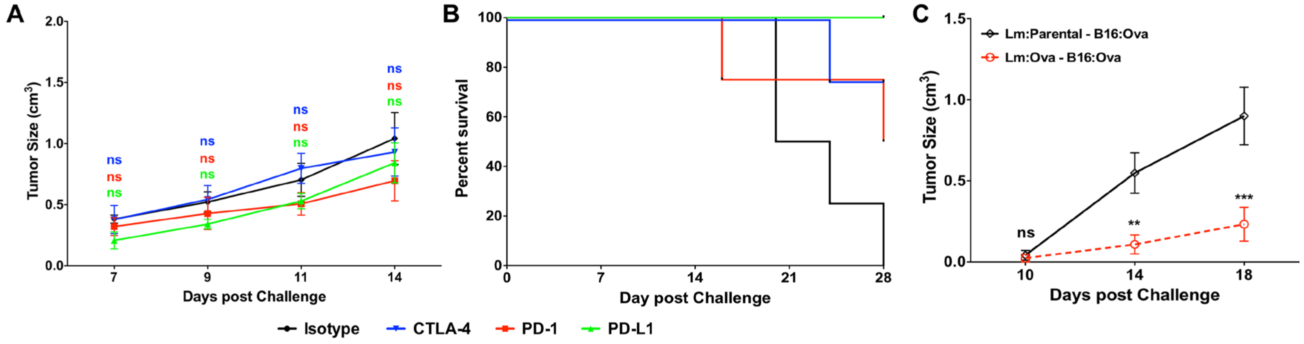

Supplementary Figure 4: Checkpoint blockade inhibitors do not significantly reduce tumor size in unvaccinated mice. Tumor size (**A**) and survival (**B**) in unvaccinated mice given either isotype (black), α-CTLA-4 (blue), α-PD-1 (red), or α-PD-L1 (green). (**C**) B16F10: OVA tumor sizes in female mice 82 days post vaccination with either Lm: Parental or Lm: OVA (n = 13 per group). Tumor sizes were analyzed by two-way ANOVA with Bonferroni post-test; $^{*}p \leq 0.05$, $^{**}p \leq 0.01$, $^{***}p \leq 0.001$.; survival was analyzed by Mantel-Cox Log-rank test.
